# Promising Nanocarriers to Enhance Solubility and Bioavailability of Cannabidiol for a Plethora of Therapeutic Opportunities

**DOI:** 10.3390/molecules27186070

**Published:** 2022-09-17

**Authors:** Lucia Grifoni, Giulia Vanti, Rosa Donato, Cristiana Sacco, Anna Rita Bilia

**Affiliations:** 1 Department of Chemistry, University of Florence, Via Ugo Schiff 6, Sesto Fiorentino, 50019 Florence, Italy; 2 Department of Health Sciences, University of Florence, Viale Pieraccini 6, 50139 Florence, Italy

**Keywords:** cannabidiol, nanocarriers, solubility, bioavailability, efficacy

## Abstract

In recent years, the interest in cannabidiol (CBD) has increased because of the lack of psychoactive properties. However, CBD has low solubility and bioavailability, variable pharmacokinetics profiles, poor stability, and a pronounced presystemic metabolism. CBD nanoformulations include nanosuspensions, polymeric micelles and nanoparticles, hybrid nanoparticles jelled in cross-linked chitosan, and numerous nanosized lipid formulations, including nanostructured lipid carriers, vesicles, SNEEDS, nanoemulsions, and microemulsions. Nanoformulations have resulted in high CBD solubility, encapsulation efficiency, and stability, and sustained CBD release. Some studies assessed the increased C_max_ and AUC and decreased T_max_. A rational evaluation of the studies reported in this review evidences how some of them are very preliminary and should be completed before performing clinical trials. Almost all the developed nanoparticles have simple architectures, are well-known and safe nanocarriers, or are even simple nanosuspensions. In addition, the conventional routes of administration are generally investigated. As a consequence, many of these studies are almost ready for forthcoming clinical translations. Some of the developed nanosystems are very promising for a plethora of therapeutic opportunities because of the versatility in terms of the release, the crossing of physiological barriers, and the number of possible routes of administration.

## 1. Introduction

*Cannabis sativa* L. (Cannabaceae), commonly known as hemp, is a plant that has been used for food, fibre, and as a medicinal herbal drug for several millennia. Probably originating from Central Asia, hemp was domesticated very early throughout Eurasia, and before its classification as a narcotic, insights concerning its medicinal use date back to The Lancet and BMJ in the late 1800s [1,2].

**Figure 1:**
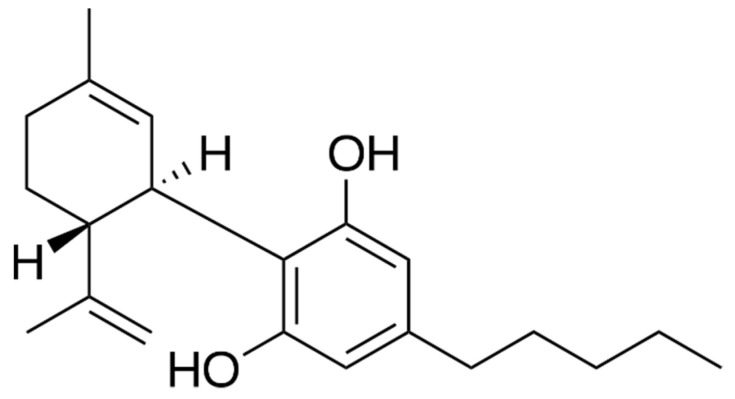
Chemical structure of CBD.

Phytocannabinoids represent the characteristic constituents of hemp, and they are widely accumulated in the glandular trichomes on the female inflorescences of *C. sativa*. They represent a unique group of isoprenoid polyketides, having a resorcinol moiety, mainly represented by cannabidiol (CBD) (Figure 1) and Δ9-tetrahydrocannabinol (THC) [3].

Nowadays, the domesticated forms of *C. sativa* have different CBD and THC contents, which are used to discriminate its industrial, medicinal, or recreational end uses, and to classify the species in the subtaxa.

Accordingly, three chemotypes based on the THC/CBD ratio are now available: the first having high-content THC (CBD/THC ratio: 0.00–0.05), a second one with high-content CBD (CBD/THC ratio: 15–25), and a third one with a CBD/THC ratio of 0.5–3.0 [4].

The pharmacological and toxicological interest in phytocannabinoids is due to the presence in the human body of the endocannabinoid system, which supports many physiological processes and has regulatory effects in a plethora of apparently distinct disorders, including neurological, cancer, and cardiovascular diseases [5].

The potential therapeutic activity of phytocannabinoids is principally related to their association with G-protein-coupled cannabinoid receptors denominated type 1 (CB1) and type 2 (CB2). However, other imperative networks include endogenous cannabinoids, such as anandamide, proliferator-activated receptor, transient receptor potential channels, and various orphan receptors (i.e., GPR55 and GPR18) [6].

Up to now, the United States Food and Drug Administration (FDA), European Medicines Agency (EMA), and other regulatory agencies have approved a few *C. sativa*-derived drugs. Sativex is an oral spray dosage form that is currently legal for sale in over 25 countries. It contains both CBD and THC as the primary constituents (a blend of extracts high in THC and CBD in a roughly 1:1 ratio), and it is produced from natural cannabis without synthetic ingredients. The drug’s primary application is the managing of severe pain, and principally pain caused by sclerosis. On 17 February 2016, the European Commission granted an orphan designation (EU/3/16/1621) for delta-9-tetrahydrocannabinol and cannabidiol from extracts of the *Cannabis sativa* L. plant for the treatment of glioma [7]. Some formulations are nowadays on the market under the brand names of Marinol and Dronabinol, both sold in solid dosage form (capsules) and containing as the active principle the synthetic form of THC. The principal uses of these medicines is to limit chemotherapy-induced nausea and to treat AIDS-induced anorexia and other related symptoms. Epidiolex is a further formulation on the market, sold as a liquid dosage form, and CBD (only botanically derived) is the active principle. It is indicated for the treatment of Lennox–Gastaut syndrome and Dravet syndrome in patients from two years of age. It is also used to treat tuberous sclerosis complex with other epilepsy treatments in patients aged two years and above. Both are very rare diseases, and accordingly, Epidiolex was designated as an “orphan medicine” by the EMA [8].

## 2. CBD: A Promising Drug Molecule with Biopharmaceutical Issues

CBD is the first isolated cannabinoid that dates back to the late 1800s, and it was obtained as a crystalline molecule by acetylation from a crude narcotic red oil distilled from the resin of Indian hemp [9]. Indeed, over 500 different compounds have been identified from *Cannabis sativa* [10]. In particular, about 130 cannabinoids have been identified, about 120 terpenoids, 42 noncannabinoid phenolics, 34 flavonoids, 3 sterols, and 2 alkaloids. Cannabinoids are in the leaves, flowers, resin, stembarks, and roots. Noncannabinoid phenols, including stilbenoids, phenanthrenes, lignans, and phenolic amides, are present in the leaves, flowers, stems, hemp pectin, resin, fruit, seeds, and roots. Terpenoids represent the second largest class of constituents, and they are also responsible for the typical smell of the plant. They include monoterpenes and sesquiterpenes, which are typical essential-oil constituents, in addition to diterpenes and triterpene. They are distributed in the leaves, flowers, stembarks, and roots. The leaves, flowers, seeds, and fruit contain flavonoids, while sterols and alkaloids are distributed in the stembarks, roots, and leaves. A very high content of oil, represented by saturated and unsaturated fatty acids and their glycerol esters, is contained in the seeds [11].

Among the cannabinoids, THC and CBD represent the principal constituents and the main psychoactive and non-psychoactive cannabinoids, respectively.

In recent years, the interest in CBD has increased because of the lack of psychoactive properties and its easy availability. Indeed, it can be obtained by purifying *Cannabis sativa* L. extracts, or by chemical synthesis. The interest is also due to the astonishing plethora of possible uses in therapy.

CBD has an affinity with more than 65 molecular targets [12]. In particular, it acts as a serotonin 1A receptor (5-HT1A), regulating the cannabinoid-related receptors G-protein-coupled receptor 55 (GPR55) and transient receptor potential vanilloid 1 (TRPV1). In addition, CBD acts on the type 1 equilibrative nucleoside transporter (ETN1), fatty acid-binding protein (FABP), nuclear factor erythroid 2-related factor 2 (NRF2), voltage-activated T-type calcium channels, adenosine and glycine receptors, mu and delta opioid receptors, and voltage-dependent anion channel 1 (VDAC1), among the most relevant.

Recent reports suggest that CBD elevates the levels of the endocannabinoid anandamide (AEA) when administered to humans, suggesting that phytocannabinoids target the cellular proteins involved in endocannabinoid clearance. On the one hand, fatty acid-binding proteins (FABPs) are intracellular proteins that mediate AEA transport to its catabolic enzyme fatty acid amide hydrolase (FAAH). On the other hand, CBD inhibits the degradation, via FAAH, and uptake of endocannabinoids, resulting in an increase in endocannabinoid–receptor binding. Once it is inside the cell, anandamide is broken down by FAAH, a metabolic enzyme, as part of its natural molecular lifecycle. However, CBD interferes with this process by reducing anandamide’s access to FABP transport molecules, and by delaying the endocannabinoid passage into the cell’s interior [13].

Although CBD has little binding affinity for either of the two cannabinoid receptors (CB1 and CB2) [14], CBD modulates several noncannabinoid receptors and ion channels. CBD also acts through various receptor-independent pathways; for example, by delaying the “reuptake” of endogenous neurotransmitters (such as anandamide and adenosine), and by enhancing or inhibiting the binding action of certain G-protein-coupled receptors.

**Figure 2:**
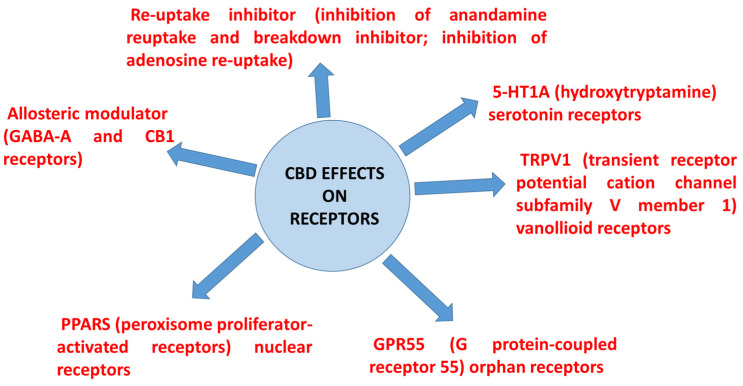
CBD effects on some receptors in addition to CB1 and CB2 receptors.

Figure 2 reports on the manifold therapeutic effects of CBD due to its interactions with diverse receptors. At high concentrations, CBD directly activates the 5-HT1A (hydroxytryptamine) serotonin receptor, thereby conferring an antianxiety effect. This G-protein-coupled receptor is implicated in a range of biological and neurological processes, including (but not limited to) anxiety, addiction, appetite, sleep, pain perception, nausea, and vomiting [15]. CBD also binds to TRPV1 receptors, which also function as ion channels [16]. TRPV1 is well known to mediate pain perception, inflammation, and body temperature. Whereas CBD directly activates the 5-HT1A serotonin receptor and several TRPV ion channels, some studies indicate that CBD functions as an antagonist that blocks or deactivates another G-protein-coupled receptor known as GPR5, which is widely expressed in the brain, and especially in the cerebellum. GPR5 is involved in modulating blood pressure and bone density, among other physiological processes. Finally, GPR55 is expressed in various types of cancer, and when activated, it also promotes cancer cell proliferation [17].

Furthermore, CBD also exerts an anticancer effect by activating peroxisome proliferator-activated receptors (PPARs) that are situated on the surface of the cell’s nucleus. The activation of the receptor known as PPAR-gamma has an antiproliferative effect, as well as an ability to induce tumour regression in human lung cancer cell lines. PPAR-gamma activation degrades amyloid-beta plaque, which is a key molecule linked to the development of Alzheimer’s disease. Therefore, CBD can play a clinical role in patients with Alzheimer’s disease. PPAR receptors also regulate the genes that are involved in energy homeostasis, lipid uptake, insulin sensitivity, and other metabolic functions. Diabetics, accordingly, may benefit from a CBD-rich treatment regimen [18].

CBD functions as an anandamide reuptake and breakdown inhibitor, thereby raising the endocannabinoid levels in the brain’s synapses. Enhancing the endocannabinoid tone via reuptake inhibition may be a key mechanism whereby CBD confers neuroprotective effects against seizures, as well as many other health benefits [13].

CBD’s anti-inflammatory and antianxiety effects are in part attributable to its inhibition of adenosine reuptake. By delaying the reuptake of this neurotransmitter, CBD boosts the adenosine levels in the brain, which regulates the adenosine receptor activity. A1A and A2A adenosine receptors play significant roles in cardiovascular function, regulating the myocardial oxygen consumption and coronary blood flow. These receptors have broad anti-inflammatory effects throughout the body [19].

CBD is also an allosteric receptor modulator, which means that it can either enhance or inhibit how a receptor transmits a signal by changing the shape of the receptor. CBD interacts with the GABA-A receptor in a way that enhances the receptor binding affinity for its principal endogenous agonist, gamma-aminobutyric acid (GABA), which is the main inhibitory neurotransmitter in the mammalian central nervous system. Hence, CBD reduces anxiety by changing the shape of the GABA-A receptor in a way that amplifies the natural calming effect of GABA [20].

Moreover, CBD is a “negative allosteric modulator” of the CB1 receptor, which is mainly present in the brain and central nervous system. CBD does not bind to the CB1 receptor directly, as THC does; however, it interacts allosterically with CB1, modifying the shape of the receptor in a way that weakens the ability of THC to bind to CB1.

As a negative allosteric modulator of the CB1 receptor, CBD-rich products with little THC can convey therapeutic benefits without having a euphoric or dysphoric effect [21]

According to all these studies, many properties have been reported for CBD, and namely, antiepileptic and anxiolytic properties, together with the prevention of Parkinson’s disease, schizophrenia and psychosis, Alzheimer’s disease, [22,23,24], and numerous kinds of tumours [25]. In addition, CBD has pronounced anti-inflammatory activity [26], potent antimicrobial activities [27], and great effectiveness in skin-related diseases [28], with a worthy safety profile [29].

However, the numerous promising clinical uses of CBD, and substantial issues, including the low bioavailability, variable pharmacokinetic profiles, and poor stability, limit the success of CBD as a medicine. CBD has very low water solubility (12.6 mg/L) and high lipophilicity (logP of 6.3), where logP is the logarithm of a drug’s partition coefficient between n-octanol and water. In addition, CBD has weak acidic properties (pKa 9.1), a melting point of 67 °C, and a molar mass of 314 g/mol. Therefore, CBD can be classified as a Class II drug of the Biopharmaceutics Classification System (BCS), which makes it a poorly water-soluble and highly permeable drug, which is eliminated by metabolism. The literature describes the presystemic metabolism as a result of phase I oxidation, which is mainly due to CYP3A4 and CYP2C19, and phase II glucuronidation via UGT1A9 [30,31]. Consequently, according to the World Health Organization, the CBD oral bioavailability is approximately only 6% [32].

Due to the highly lipophilic nature, CBD is generally supplied as an oily or alcoholic formulation, either in soft-gel capsules, liquid solution, sublingual drops, or as an oromucosal spray [33]. In addition, studies investigating the oral and oromucosal delivery of CBD and THC at equimolar concentrations in humans have evidenced high inter-/intra-individual variability [34]. Finally, CBD can precipitate in the gastrointestinal tract, with a subsequent poor absorption rate.

The very low CBD oral bioavailability of CBD is due to irregular absorption because of the high lipophilicity, instability in the stomach acidic environment, and pronounced first-pass liver metabolism. The reported time to peak plasma concentration is between 1 and 4 h. After the administration of oromucosal spray (20 mg), the C_max_ was 2.4 ng/mL of CBD, and the half-life was between 1.4 and 10.9 h [35]. For the commercial preparations Epidiolex and Sativex, similar pharmacokinetic parameters are reported, but Sativex, administered as an oromucosal spray, provides a faster onset of action. Additionally, the bioavailability of inhaled CBD is much more (about 31%), but it is strongly dependent on the inhalation method and breath duration. Finally, after topical administration, an accumulation of CBD in the stratum corneum occurs without penetration to the deeper tissue layers. Indeed, although CBD has a suitable molecular weight (314.46 Da), its high logP value strongly limits transdermal delivery. A further issue of CBD is the low stability: it is easily degraded by light and auto-oxidation, and at room temperature [36].

## 3. Nanosized Strategies to Improve Biopharmaceutical Performances of Class II Drugs

According to the scientific data reported in Paragraph 2, CBD is a typical drug of the Class II Biopharmaceutics Classification System (BCS), which is widely accepted today in the academic, industrial, and regulatory worlds [37].

In the last decade, the studies to enhance the solubility of Class II drugs through delivery strategies have increased because almost 90% of the new drugs are poorly soluble compounds. The bioavailability of Class II drugs is related to the dissolution rate, which is ultimately related to the solubility. Consequently, an increased solubility produces an improved bioavailability in vivo. Many strategies can be used to optimise the dissolution rate, including the production of amorphous powders, and supramolecular complexes with cyclodextrins, micronisation, and nanonisation [38].

Nanonisation is a process to reduce the particle size of the active pharmaceutical ingredient (API) to a nanometre size range. In fact, according to the Noyes–Whitney equation, a decrease in the particle size of a drug results in an increase in its surface area, and thus the dissolution rate will increase proportionally, resulting in the better absorption of poorly soluble drugs. Additionally, the API could be formulated using nanoscale carriers, including vesicles and micelles, lipid nanoparticles, nano- and microemulsions, and polymeric and inorganic nanoparticles. These nanosystems aid in preventing drugs from being tarnished in the gastrointestinal region, and they help the delivery of Class II drugs to their target locations. A main characteristic of these nanodrug delivery systems is the high versatility in their administration routes, including the parenteral, oral, nasal, pulmonary, ocular, and transdermal routes [39].

The choice of the nanometre range for drug delivery loaded with Class II drugs is also linked to their ability to either cross biological barriers themselves, or to allow loaded drugs to cross them. This is a key factor for some specialised barriers, such as barriers between the blood and neural tissues, and in particular, the blood–brain barrier (BBB), which are very difficult to cross by drugs because of their function for maintaining the homeostasis of the brain by regulating the chemical environment, immune cell transport, and entry of xenobiotics. The BBB represents the most important factor limiting the development of drugs for the central nervous system, and it is characterized by relatively impermeable endothelial cells with tight junctions, enzymatic activity, and active efflux transport systems. The tight junctions formed by brain microvascular endothelial cells regulate paracellular transport, whereas transcellular transport is regulated by specialised transporters, pumps, and receptors. Nanocarriers can open the tight junctions between endothelial cells, transcytosis through the endothelial cell layer, and endocytosis by endothelial cells, releasing the drug inside the cell. Furthermore, coating agents, such as polysorbates, inhibit the transmembrane efflux systems (i.e., P-glycoprotein). Various novel drug delivery systems, such as liposomes, microspheres, polymeric nanoparticles, lipid nanoparticles, and inorganic systems, have been proposed to overcome the limitations imposed by the BBB [40].

## 4. Nanosized Drug Delivery Systems Loaded with CBD

Nanosized drug delivery systems, which received their appellation from their nanometre (nm) size (generally from a few nanometres up to some hundreds of nanometres), have increased considerably in the literature and market over the last two decades. Two approaches are generally reported: nanosuspensions, and nanovectors, which are characterised by the huge loading properties of the drug and are considered easily taken up by cells [41].

Nanosuspension formation can improve the solubility, permeability, and ultimately, the bioavailability. An increase in the surface area to volume ratio will result in increased cellular uptake due to the 100-fold size reduction [42]. Nanosuspensions present a substantial enhancement of the in vivo bioavailability, they are easy to prepare, and it is easy to scale up the developed nanoformulations [43].

Nanosuspensions are formulated by dispersing insoluble drugs in aqueous media in the presence of appropriate excipients, followed by particle size reduction in a media mill, wherein the drug particles are broken down via bead collision. They generally need surfactants or hydrophilic polymers during the formulation to impede agglomeration and cake formation. The advantages after oral or other routes of administration are numerous, and they are represented by fast dissolution rates with a consequently improved bioavailability; even the limits are represented by the absence of vectors, with a consequent lack of controlled release, passive or active targeting, and limited protection from physical or chemical degradation [44].

In addition, nanovectors represent very encouraging tools because, like nanosuspensions, they are characterised by a huge surface area to volume ratio, and their ability of encapsulation efficiency allows for an extended circulation time, reduced clearance rates, increased physical and chemical stabilities, improved cell uptake, and an optimised pharmacokinetic profile [30]. A further auspicious feature of using nanovectors is to deliver the drugs to the target site via active or passive methods due to the flexibility of the functionalisation of their surface with hydrophilic polymers or targeting ligands (i.e., peptides or small molecules, such as folic acid or antibodies). Targeting confers an increased selectivity towards the targeted cells/tissues/organs to improve their therapeutic efficacy [44].

**Figure 3:**
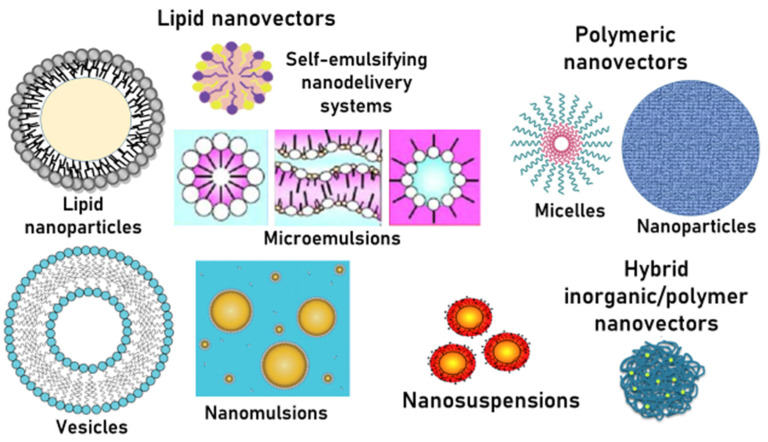
Nanosuspensions and polymeric, lipid-based, and hybrid nanocarriers.

According to the nature of the nanomaterials, the nanovectors can be classified as polymeric, lipid-based, and inorganic nanocarriers (Figure 3). Polymeric nanovectors can be derived from natural (polysaccharides and proteins) or synthetic polymers, and they can be further subclassified into biodegradable and non-biodegradable polymers. The lipid-based nanovectors include microemulsions and nanoemulsions, vesicles, solid lipid nanocarriers, and nanostructured lipid carriers. Lastly, the inorganic nanocarriers include many nanostructures, including quantum dots, carbon nanotubes, and gold, magnetic, and silica nanoparticles [39].

### 4.1. Nanosuspensions

A unique and very recent study investigated the in vitro and in vivo performances of a CBD nanosuspension after oral and intramuscular administration, comparing the nanosuspensions with unformulated CBD and a CBD commercial oily formulation. Tween 80 (0.50%, *w*/*v*) was selected as a stabiliser to produce the CBD nanocrystals by antisolvent precipitation. The CBD displayed a particle size of 141.7 ± 1.5 nm, a polydispersity index (PDI) of 0.18 ± 0.01, and a ζ-potential of −25.73 mV, and it was freeze-dried using bovine serum albumin as a cryoprotectant. The nanocrystals were physically and morphologically characterised. The in vitro dissolution profiles of the CBD raw material, the physical mixture of CBD and Tween 80, and the CBD-freeze-dried nanosuspension were evaluated. The CBD in the physical mixture had a better release than the CBD raw material, reaching a cumulative dissolution rate of 42.91% after 15 min. In comparison, after the same time interval, the CBD freeze-dried powder reached 91.57%. In vivo pharmacokinetic studies evidenced that the nanosuspension after the oral and intramuscular administrations was significantly superior to the oily formulation. In addition, the AUC_0–24h_ and C_max_ of the CBD by the intramuscular injection of a nanosuspension increased significantly compared with the oral administration of the nanosuspension. The C_max_ values were 239.41 ± 16.92, 151.40 ± 35.78, and 135.94 ± 38.15 ng/mL, respectively, for the nanosuspension after intramuscular administration, the oral administration of the nanosuspension, and the oral administration of the oily conventional formulation [44].

The CBD nanosuspensions were prepared using flash nanoprecipitation and lecithin, or hydroxypropyl methylcellulose acetate succinate (HPMCAS), using a 1:1 mass ratio of CBD/stabiliser. The lecithin-stabilized nanoparticles had a size of 176 ± 11 nm and a PDI of 0.18 ± 0.04, while the HPMCAS-stabilized nanoparticles were 181 ± 5 nm, with a PDI of 0.13 ± 0.08. A control formulation of CBD without a stabiliser displayed large aggregates with a size of 919 ± 183 nm, and a PDI of 0.76 ± 0.14. Trehalose was used as a cryoprotectant for the CBD−lecithin nanoparticles, and hydroxypropyl methylcellulose as a cryoprotectant for CBD−HPMCAS nanoparticles. Both the lyophilized nanoformulations did not display the melting endotherm corresponding to crystalline CBD, which indicates the amorphization of CBD.

The coencapsulation of Fe_3_O_4_ was investigated to evaluate the in vitro release profile. The resulting nanoparticle formulations contained a 1:1:1 mass ratio of CBD/stabilizer/Fe_3_O_4_ colloids. The CBD−lecithin−Fe_3_O_4_ nanoparticles displayed a size of 156 ± 10 nm, with a PDI of 0.17 ± 0.05, while the CBD−HPMCAS−Fe_3_O_4_ nanoparticles were 287 ± 11 nm, with a PDI of 0.22 ± 0.04. The in vitro release profiles of the CBD−lecithin−Fe_3_O_4_ and CBD−HPMCAS−Fe_3_O_4_ nanoparticles were compared to bulk crystalline CBD and bulk amorphous CBD. Both nanoformulations displayed rapid dissolution kinetics in the simulated intestinal medium. The lecithin-stabilised nanoparticles exhibited immediate complete burst release, while the HPMCAS stabilised particles displayed an initial rapid release (80%), followed by a gradual increase. Both nanoformulations displayed a six-fold improvement in the dissolution compared with the crystalline CBD, which showed partial dissolution (45%) over the 6 h in vitro assay. In contrast, the bulk amorphous CBD exhibited a limited dissolution (20%): less than half of the crystalline CBD. Nanoformulations containing coencapsulated Fe_3_O_4_ colloids were investigated to evaluate the effect of the colloids on the release kinetics of CBD. The study evidenced that the release profile did not depend on the levels of coencapsulated Fe_3_O_4_ [45].

### 4.2. Polymeric Nanocarriers

#### 4.2.1. Micelles

Two studies on CBD nanomicelles were developed for topical delivery, one for the cutaneous route [46], and the other for ocular administration [47]. The first contribution reported the development of original nanocomposite cryogels for the sustained topical delivery of CBD. First, polymeric core–shell micelles were prepared and loaded with CBD. The nanomicellar formulation was then embedded in a cryogel carrier obtained via UV-assisted cryotropic gelation using hydroxyethyl cellulose. Pluronic F127 was selected to develop block copolymer micelles, with final concentrations of the polymer and CBD of 30 and 10 g·L^−1^, respectively, corresponding to 25% drug loading. The characterisation of the micelles was performed by DLS measurements, evidencing the formation of a dominant population of small micelles with a hydrodynamic diameter of 21 nm, with relatively narrow dispersity and an extra less intensive peak (size = 173 nm), suggesting the existence of larger aggregates. Next, the micellar solution was mixed with an aqueous solution of hydroxyethyl cellulose and cross-linking using different agents and photoinitiator H_2_O_2_. The cryogel was obtained from the mixture after homogenisation, cooling at a temperature of −20 °C for 2 h, and irradiation with UV light for 2 min. The pure cryogel and the cryogel loaded with the CBD formulation were evaluated by scanning electron microscopy to analyse the pores, wall thickness, and surface decorated with the nanosized CBD-loaded polymeric micelles. The nanocomposites were soft and tear-resistant upon gentle handling. The release of CBD from these nanocomposites had no burst effect and a pronounced release of CBD for the studied period of 24 h. Finally, the ability to retain the tumour inhibitory effects of CBD in the nanocomposite was tested using two cell lines (cell lines MJ and T-24) [46].

A further study developed mucoadhesive mixed polymeric micelles made of chitosan and poly (vinyl alcohol) (PVA) backbones graft-hydrophobized with short poly (methyl methacrylate) blocks loaded with CBD using free radical graft polymerisation. Then, the CBD-loaded mixed nanomicelles were physically stabilised by the ionotropic crosslinking of the chitosan domains with sodium tripolyphosphate and spray-drying. These nanomicelles display a CBD loading capacity of 20% *w*/*w*, and sizes of 100–200 nm, with a spherical morphology, evaluated by cryogenic-transmission electron microscopy. The nanoformulation was stabilised by noncovalent cross-linking, maintaining the size of the nanoformulation at less than 200 nm, which is appropriate for enhanced mucosal delivery. A human corneal epithelial cell line was used as an in vitro model to simulate the eye’s outer surface. Before and after the cross-linking process, the cell compatibility was very high. Finally, the ability of CBD to cross the human corneal-epithelial-cell monolayers (an epithelium model in vitro) was a crucial step in ensuring efficient ocular drug delivery. To estimate the integrity of the human corneal-epithelial-cell monolayers, the TEER values were measured over 14–21 days. The permeability evaluation was investigated using two corneal-epithelium models with air–liquid and liquid–liquid interfaces. The findings showed that, in both models, the permeation was very good and dependent on the concentration. Under the liquid–liquid-interface conditions, fresh 0.03% cross-linked mixed PMs displayed a P_app_ of 39 ± 7 × 10^−7^ cm/s, while for the underneath air–liquid interface, the P_app_ value was 24 ± 5 × 10^−7^ cm/s [47].

#### 4.2.2. Nanoparticles

Poly-lactic-co-glycolic acid (PLGA) nanoparticles and PLGA nanoparticles coated with chitosan were developed. They were characterised by smooth surfaces and a spherical morphology, having sizes of 192.90 ± 2.41 and 287.20 ± 0.90 nm, respectively, and PDIs of 0.041 ± 0.027 and 0.134 ± 0.025, respectively. The ζ-potential of the nanoparticles was −6.270 ± 0.927 mV, while the coated nanoparticles presented a value of +3.370 ± 0.158 mV. The encapsulation efficiency of the CBD-loaded nanoparticles was 70.31 ± 0.69%, while that of the coated ones was 78.52 ± 0.82%. Both types of nanoparticles were stable for 5 weeks at 4 °C. The release of CBD from the two kinds of nanoparticles was evaluated using different pH values representing different environments, namely, pH 5.0 (lysosome) and pH 6.5 (tumour microenvironment and urine environment). A typical biphasic release behaviour was found, including an initial burst release up to the first 15 h, followed by a continuous slow-release mode, lasting up to 110 h. The PLGA nanoparticles evaluated at a pH of 6.5 showed an initial burst rate of 34.64 ± 2.22% within 15 h, and the cumulative-release rate reached 49.85 ± 2.76% at 110 h. At a pH of 5.0, the burst-release rate was 50.55 ± 2.02%, and at 110 h, the cumulative-release rate reached 69.07 ± 2.46%. The PLGA-coated nanoparticles produced a slow drug release, while the cumulative-release rate was less than 20% (pH 6.5: 13.54 ± 0.40%; pH 5.0: 15.67 ± 0.78%), probably due to the chitosan. In addition, the chitosan-coated nanoparticles significantly enhanced the adhesion to the mouse bladder wall, and the binding efficiency of the mucin-to-chitosan–PLGA nanoparticles reached 97.04% ± 1.90%. The nanoparticles’ uptake ability was higher for the coated nanoparticles after 2 and 6 h of incubation. As a final step of the study, the in vitro cytotoxicity of the CBD-loaded nanoparticles was evaluated in T24 cells and SV-HUC-1 cells. The cell viability of the pure CBD in both cell lines had no significant toxicity at any of the tested concentrations, including at 50 µM. The blank nanoparticles had no significant cytotoxicity (cell viability of more than 90%) against either cell line at a polymer concentration of 10–50 µM. Truly, at the highest polymer concentration, the cell viability still exceeded 90%, indicating the high safety of the drug delivery system. In addition, both types of nanoparticles could inhibit the proliferation of T24 cells in a time- and concentration-dependent manner, without causing damage to NBC SV-HUC-1. The study evidenced that the therapeutic effect of nanoparticles coated with chitosan was not as good as the uncoated ones, and worse than free CBD, probably due to the slowed-down release of the CBD [48].

In a further study, PLGA nanoparticles loaded with CBD were formulated for the intraperitoneal administration of chemotherapeutics. The CBD-loaded nanoparticles had a size of about 240 nm, with a spherical shape, and a negative ζ-potential (−16.6 ± 1.2 mV). The drug loading of the nanoparticles was 140.20 ± 6.25 µg CBD/10 mg nanoparticles, and the encapsulation efficiency was high (95.23% ± 3.30%). A controlled CBD release over 96 h was detected in the in vitro studies, with 100% of the CBD released at the end of the experiment. Within the first hour, a high burst effect was observed (about 35% of the CBD was released). When the CBD release profile fitted with the zero-order kinetics (r = 0.952), the release rate was 21.6 µg day^−1^/10 mg nanoparticles. The short-term stability at 5 °C over three months was used to assess the physical and chemical stabilities of the formulation. The nanoparticle internalization was tested in SKOV-3 epithelial ovarian cancer cells. No internalization occurred during the first 30 min; it started to become significant after 2 h of incubation, increasing up to 4 h. Significant higher uptake was not detected after 6 and 8 h. Finally, it was assessed that the CBD antiproliferative activity in ovarian cancer cells was preserved after encapsulation. Lower IC_50_ values were found for the nanoparticles compared with free CBD, although both induced the expression of PARP, indicating the onset of apoptosis [49].

In a further study, zein/whey protein (WP) nanoparticles were developed and loaded with CBD. Whey proteins principally constituted of β-lactoglobulin (70%) and other proteins (lactoferrin, bovine serum albumin, and α-lactalbumin) can be absorbed by zein nanoparticles through the generation of an interpolymeric system, preventing the aggregation of nanoparticles. The optimised nanoformulation contained zein/whey protein with a ratio of 1:4, and the nanoparticles were loaded with CBD (200 μg/mL). The zein nanoparticles had a size of ca. 70 nm. After blending with whey protein, the size increased to 140–160 nm, but the PDI decreased: 0.18 for the zein nanoparticles, and 0.06–0.1 for the zein/whey protein nanoparticles. The ζ-potential was positive in the zein nanoparticles (ca. +37 mV) and negative (ca. −40 mV) in the zein–WP nanoparticles. The CBD encapsulation efficiency of the zein nanoparticles was 77%, while the CBD encapsulation efficiency of the zein/whey protein nanoparticles was 89%. Conversely, the loading capacity of the zein nanoparticles was 3.2%, while it was only 0.75% for the zein/whey protein nanoparticles. The solubility of pure CBD in water was ca. 0.39 µg/mL (less than those reported in the literature), while the CBD solubility in the zein nanoparticles was ca. 170 µg/mL. Accordingly, the solubility was significantly increased to ca. 196 µg/mL for the zein/whey protein nanoparticles (*p* < 0.05). The increased solubility was also due to the amorphous state of the encapsulated CBD. The zein/whey protein nanoparticles showed excellent storage stability (4 °C, dark), and they effectively protected the CBD degradation against heat and UV light. A pharmacokinetic study was carried out in male Sprague Dawley rats to compare the performances of zein/whey protein nanoparticles and pure CBD after an oral administration of 40 mg/kg of CBD. The C_max_ and AUC–∞ were, respectively, 0.232 μg/mL and 1.657 μg/mL/h for the pure CBD, confirming the poor absorption of CBD in vivo. By contrast, the administration of CBD encapsulated in zein–WP nanoparticles reflected an increased C_max_ and AUC0–∞ (0.466 μg/mL and 2.912 μg/mL·h, respectively), with about 2-fold and 1.75-fold improvements, respectively. Additionally, a lower T_max_ (2 h) occurred with the nanoparticles compared with the free CBD (4 h). Finally, CBD was still detectable in the plasma at 12 h after the administration of the nanoparticles [50].

### 4.3. Lipid-Based Formulations

#### 4.3.1. Nanoemulsions, Microemulsions, and Self-Emulsifying Nanodrug Delivery Systems

In a recent study, different nanoemulsions were developed and optimised to obtain size droplets of 120 nm, and they were loaded with CBD. The developed nanoemulsions were prepared using poloxamer 188 as a stabiliser and different oils, including medium-chain triglycerides, trimyristin (a triglyceride), soybean oil, and rapeseed oil. Surprisingly, a high CBD load (more than 40%) was observed for all the emulsions. In particular, the emulsions based on medium-chain triglycerides and trimyristin displayed the highest CBD loading (ca. 70) with respect to the soybean oil (ca. 49%) and rapeseed oil (ca. 43%). The elevated CBD loading in trimyristin, a solid crystalline lipid, could be related to the supercooled melt after processing into the colloidal state while remaining in the liquid form [51].

An additional study compared the pharmacokinetic parameters of CBD formulated in sesame oil (CBD 2% *w*/*w*) vs. a self-nano-emulsifying drug delivery system (CBD 3% *w*/*w*), which, after gentle agitation in the aqueous phase, spontaneously formed nanodroplets with a size of less than 50 nm. The nanoformulation consisted of ethanol (23% *w*/*w*), soy phosphatidylcholine (4% *w*/*w*), sesame oil (12% *w*/*w*), Tween 20 (20% *w*/*w*), Span 80 (19% *w*/*w*), and Kolliphor RH40 (22% *w*/*w*). The conventional formulation and nanoformulation were given orally at a CBD dose of 15 mg/ kg. CBD was also administered intravenously as a bolus at 1 mg/kg dose. The intravenous bolus administration of CBD was used to calculate the clearance (1677 ± 230 mL/h/kg) and steady-state volume (*V*ss) of the distribution (7673 ± 1270 mL/kg). The oral administration of CBD in the sesame oil apparently produced 2-fold and 4-fold increases compared with the nanoformulation in the C_max_ (561 ± 116 vs. 266 ± 36 ng/mL) and AUC_inf_ (2713 ± 552 vs. 727 ± 64 h × ng/mL). After the intravenous administration, the AUC_inf_ (h × ng/mL) and K_el_ (h^−1^) were 605 ± 110 and 0.2 ± 0.06, respectively. The T_max_ values found for the two rat groups were different: 4 h for the sesame oil and 1.5 h for the nanoformulation. The sesame oil displayed a larger range (2−4 h) compared with the nanoemulsion (1.5−2 h). The K_el_ values of the two formulations were similar (0.03 ± 0.03 and 0.3 ± 0.06, respectively) and were comparable to that obtained with the CBD intravenous administration (0.2 ± 0.06) [52].

A further study by the same authors aimed to investigate the effect of the long-chain and medium-chain lipid components in self-emulsifying nanodrug delivery systems on the oral absorption of selected molecules coadministered with CBD. Stable oil-in-water nanoemulsions were produced, showing a particle size of 50 nm or less, and a PDI of 0.3 or less. Four nanoformulations were selected, two based on sesame oil or Miglyol 812N (SNEDDS I), and two based on cocoa butter or tricaprin (SNEDDS II). The absorption kinetics of CBD after oral administration were evaluated in a rat model at a 15 mg/kg dose. CBD–SNEDDS type I had similar in vivo pharmacokinetic parameters. The C_max_ values (ng/mL) were 137 ± 13 and 101 ± 13, respectively, for the nanoformulation containing sesame oil or Miglyol 812N. The AUC_inf_ (h × ng/mL) values were 611 ± 38 and 579 ± 61, respectively, and the T_max_ (h) values were very similar (i.e., 1.08 (0.67−1.5) and 1 (0.67–1.5), respectively). Finally, the K_el_ (h^−1^) did not differ between the formulations (0.1 ± 0.02). Although there was a trend in favour of the sesame oil SNEDDS formulation, the increases in the AUC and C_max_ were not statistically significant. CBD–SNEDDS type II resulted in a significant difference in the AUC and C_max_ values of 1.6-fold and 1.7-fold, respectively, in favour of the cocoa butter SNEDDS. The C_max_ (ng/mL) values were 458 ± 7 and 261 ± 58 for the cocoa butter and tricaprin nanoformulations, respectively, the AUC_inf_ (h × ng/mL) values were 2864 ± 161 and 2041 ± 211, respectively, the T_max_ (h) values were 6 (5–7) and 5 (5–6), respectively, and the K_el_ (h^−1^) values were 0.2 ± 0.1 and 0.1 ± 0.02, respectively [53].

In another study, a novel nanoemulsion loaded with CBD was developed using vitamin E acetate, ethanol, Tween 20, and distilled water (1.7/3.8/70/24.5 *w*/*w*%, respectively). The particle size was 35.3 ± 11.8 nm. The nanoemulsion loaded with CBD (50 mg/kg) and an oil formulation (CBD: 100 mg/kg, control) were orally administered to rats, and the blood samples were collected over time. Moreover, the two formulations were orally administered to bile-fistulated rats, and the pharmacokinetic profiles of the CBD were also evaluated. The mean T_max_ of the CBD of the nanoemulsion was shortened significantly by a factor of 3 when compared with the T_max_ of the CBD oily formulation (2.40 versus 8.00 h, respectively, *p* < 0.001). The AUC0-∞/dose increased by 65% compared with the CBD oily formulations (from 0.272 ± 0.045 to 0.448 ± 0.087 h L/kg). In addition, after the oral administration of the CBD oily formulation, the AUC0-∞/dose and C_max_/dose were significantly reduced by 27 and 23 times (*p* < 0.05 and *p* < 0.01, respectively), respectively, in bile-fistulated rats compared with the untreated rats. Accordingly, CBD oily formulations need bile-mediated micelle formation for absorption. Conversely, after the oral administration of the CBD-loaded nanoemulsion, the pharmacokinetic parameters were not significantly different between the untreated and bile-fistulated rats [54].

In a further study, a nanoemulsion was formulated using 15 *w*/*w*% soybean oil and 1.5 *w*/*w*% quillaja saponin, loaded with ca. 10 mg/g of CBD. The optimised nanoemulsion showed a droplet size of about 120 nm, and a ζ-potential of about −30 mV. The developed nanoemulsion was stable after 6 weeks of storage under ambient and diverse stress conditions due to increased or decreased temperature, dilution, and carbonation. Conversely, pH values less than 2, and salt concentrations greater than 100 mM, disrupted the nanoemulsion [55].

Finally, a CBD-loaded O/A microemulsion was developed and formulated as a microemulgel. The microemulsion was prepared with water (66%), Solutol HS 15 (20%), Transcutol P (9%), isopropyl myristate (5%), and 1% *w*/*w* CBD. The size of the globules was 35 ± 2 nm, with a PDI of 0.23 ± 0.02, and these values did not change after loading the CBD or after the formation of the microemulgel with Sepigel 305. The value of the pH of the microemulgel was 6.56 ± 0.20. The viscosity of the microemulgel was suitable for dermatological applications (439,000 ± 4243 mPa·s). The studies of the CBD release over 24 h gave a value of 90 ± 24 μg/cm^2^. A skin-PAMPA assay showed a CBD effective permeability of 1.67 ± 0.16 ×·10^−7^ cm/s. The absorbed dose after 24 h was evaluated as 115.30 ± 16.99 µg/cm^2^. The permeability of the CBD was 3 ± 1 μg/cm^2^ when evaluated with Franz diffusion cells using rabbit ear skin. The stability of the CBD was optimum after three months of storage, and the physical stability was assessed in the same storage conditions [56].

#### 4.3.2. Lipid Nanoparticles

A study developed nanostructured lipid carriers for CBD nasal administration. CBD-loaded nanoparticles were prepared using the hot microemulsion technique. Stearic acid (1.25% *w*/*w*) was used as a solid lipid, and oleic acid (0.75% *w*/*w*) as a liquid lipid. Cetylpyridinium chloride (0.05% *w*/*w*) was used as a surfactant to impart a positively charged surface to the nanoparticles and, consequently, mucoadhesive properties; Span 20 (0.25% *w*/*w*) was the cosurfactant of the system. Both the unloaded and CBD-loaded nanostructured lipid carriers had similar sizes (285 ± 5.2 nm and 177 ± 3.1 nm, respectively). Similar results were obtained for the PDI values (0.34 ± 0.05 vs. 0.30 ± 0.02, respectively). The structure of the nanocarriers was spherical.

The CBD encapsulation efficacy was 99.99 ± 0.0001%, while the drug loading was 18.75% ± 0.0001. The nanoparticle dispersion was jelled with Pluronics thermo-reversible polymers to obtain a suitable formulation for nasal mucosa administration. The nanoparticle dispersion showed Newtonian behaviour, while the gel loaded with the nanoparticles had pseudoplastic behaviour and a thixotropic flow, with higher viscosity at 37 °C compared with 25 °C. In vitro release studies of both the nanoparticle dispersion and thermogel evidenced similar release profiles of CBD with an initial burst (about 50% of CBD in 5 min), followed by similar slow and sustained releases. To evaluate the mucoadhesive strength of the nanoparticles loaded in the gel, they were mixed with mucin solutions (0.025–5 mg/mL). The tensile-strength mucoadhesion measurements of the developed gel containing CBD-loaded nanoparticles resulted in higher mucoadhesion than the other hydrogels used as positive samples. The nanoparticles and the gel loaded with nanoparticles were administered intranasally to mice with PTX-induced neuropathic pain. In addition, the oral administration of unformulated CBD was given as a positive control. Accordingly, the CBD oral solution induced antinociception at a dose of 5 mg/kg. The nasal administration of the CBD nanoparticle dispersion produced a significant antinociceptive effect in mice with neuropathic pain (*p* < 0.001), and the activity persisted for more than 6 h. Moreover, the CBD solution after nasal administration produced antinociception (*p* < 0.001), even if the activity persisted only 1 h 30 min, producing a faster effect than the oral administration.

Finally, the gel loaded with the nanoparticles intranasally administered did not alter the mechanical allodynia (*p* > 0.05) [57].

#### 4.3.3. Vesicles

Liposomes loaded with CBD were developed using sunflower lecithin. The size was about 100 nm, and the CBD encapsulation was 20 mg/mL. The stability was assessed after storage at 4 °C for at least 3 months. The activity of the nanoformulation was evaluated in a 4-week randomised double-blinded placebo-controlled study in a spontaneous canine model of osteoarthritis. The study evidenced the efficacy of liposomal CBD (20 mg/day), which demonstrated the same efficacy as a CBD dose of 50 mg/day. Over the 4-week analysis period, the nanoformulation had a good safety profile [58].

In a further study, special nanovesicles, nanosized transferosomes, were prepared using soy lecithin, cholesterol, and polysorbate 80. Increasing the amount of polysorbate 80 improved the entrapment efficiency, with the highest entrapment efficiency of 80% obtained by adding 100 mg. The optimised nanotransferosomes were spherical and unilamellar vesicles, with a size of 130 nm, polydispersity of 0.285, and ζ-potential of −15.97 mV. The CBD stability in the transferosomes was assessed for up to 6 months at room temperature. The drug release from the CBD-loaded transferosomes was evaluated over 7 h at 37 °C. Three formulations were compared: CBD-loaded transferosomes containing 25 mg polysorbate 80, CBD-loaded transferosomes containing 50 mg polysorbate 80, and CBD-loaded transferosomes containing 100 mg polysorbate 80. An initial rapid release was observed for all three formulations, and after 7 h of release, approximately 95% of the CBD was released for the formulations containing 25 and 100 mg polysorbate 80. About 80% of the CBD was released from the transferosomes containing 50 mg of polysorbate 80.

The permeation properties of the transferosomes containing 100 mg polysorbate 80 were assessed for 5 h using a colorectal membrane. The CBD flux of the transferosomes was approximately 1.7 mg/cm^2^/h, while the flux of the unformulated CBD was 1 mg/cm^2^/h [59].

### 4.4. Hybrid Inorganic/Polymer Nanoparticles

In a further article, a new type of ZnO nanoparticles formulated in cross-linked chitosan-based patches were designed for controlled CBD delivery. The ZnO had a size of 20 nm, with a round or slightly cubical shape. To prepare the transdermal systems, chitosan water/propandiol (1:1) was mixed with various amounts of ZnO NPs, placed in a microwave oven, and lyophilised. The CBD loading was achieved by the adsorption of different amounts of 0.5 mg of CBD/mL in a 96% *v*/*v* ethanol solution. As a result, the polymeric nanoparticles were regularly dispersed in the polymeric matrix. The swelling degree of the polymeric matrix in distilled water was 55 g/g, whereas it became roughly two times lower in the simulated body fluid. The porosity of the materials was at least 70%. The nanocomposite transdermal systems released at least 20% more CBD than the hydrogel. Finally, the formulation was evaluated by 3-(4,5-dimethylthiazol-2-yl)-2,5-diphenyltetrazolium bromide (MTT) tests to determine the cytotoxicity. L929 mouse fibroblasts cultured in a pure cell culture medium were used as a control. It was evidenced that they do not cause direct or indirect cytotoxicity to L929 mouse fibroblasts cells [60].

## 5. Discussion

This review outlines some current CBD biopharmaceutical issues, and how nanocarriers can affect its solubility, release properties, stability, and bioavailability.

Seventeen CBD nanoformulations were included in this review, including nanosuspensions, polymeric micelles and polymeric nanoparticles, inorganic nanoparticles jelled in cross-linked chitosan-based patches, and numerous nanosized lipid formulations [44,45,46,47,48,49,50,51,52,53,54,55,56,57,58,59,60]. Among the lipophilic nanocarriers, nanostructured lipid carriers, vesicles, SNEEDS, nanoemulsions, and microemulsions, the latter in the form of a microemulgel using Sepigel 305 has been developed. The investigated polymers were PLGA, PLGA plus chitosan to impart mucoadhesion, and zein/whey protein. Among the lipids used to formulate the lipid nanoparticles, lecithin, diverse natural and synthetic oils, vitamin E acetate, Solutol, Transcutol, isopropyl myristate, and numerous surfactants have been used.

The basic characterisation of nanoparticles includes at least the size and PDI obtained by dynamic light scattering, morphology, architecture obtained by scanning or transmission microscopy, encapsulation efficiency (EE%), and loading capacity. Table 1 summarises the developed nanocarriers loaded with CBD and their compositions, CBD contents, CBD encapsulation efficiencies, and stability studies. Concerning the nanosuspensions, the drug loading was only calculated for one publication [44], while the EE% was not reported because the formulations were not properly nanocarriers. For nanomicelles, the EE% was not reported but the drug loading was calculated [46,47]. For nanoparticles, both the EE% and drug loading were reported [49,50], or the data were simply limited to the EE% [48]. For nanoemulsions, microemulsions, and SMEEDS, the EE% is almost 100% because of the selection of the excipients for CBD solubility, and the drug loading is always reported in the range of 1–73% *w*/*w* [51,52,53,54,55,56,57]. For vesicles, only the drug loading [58] or drug loading plus EE% were reported [59]. For hybrid nanoparticles, no data on either the drug loading or EE% are reported [60].

Only six manuscripts reported on the CBD and physical stability of the formulated nanoparticles [48,49,50,55,56,59], as described in Table 1.

The studies assessed the physical and chemical stabilities at different temperatures: 4 °C or 5 °C, and 21 °C or 25 °C. In addition, a publication reported the stability of the developed nanoformulation at 80 °C for 1 min to simulate the high-temperature short-time pasteurization process (typically, 71.5 °C for 15 s). The physical stability of 1 mL of the nanoemulsion after dilution to a volume of a standard beverage was also assessed. Finally, the physical and chemical stabilities at 80 °C for 90 min, and from UV irradiation, were found for the CBD-loaded nanoparticles made of zein plus whey protein.

Table 2 displays the data concerning the in vitro studies of the developed nanocarriers.

Nanosuspensions, polymeric and hybrid nanoparticles, nanomicelles made of Pluronic F127, a microemulsion and microemulgel, and the nanostructured lipid particles and nanovesicles made of soy lecithin, cholesterol, and polysorbate 80 were investigated for the CBD release properties [44,45,46,48,49,50,56,57,59,60]. All the studies evidenced an augmented CBD release due to increased CBD solubility and improved dissolution properties. In vitro tests using appropriate cell lines to investigate the toxicity of the nanocarriers demonstrated optimal safety profiles, as reported in five studies [46,47,49,59,60]. In addition, three studies [44,48,57] also reported good safety profiles after in vivo studies. High permeability in the cell lines [47,48,49] and good permeation of the skin [56,60] were also found in the studies.

In addition, numerous pharmacokinetic and bioavailability studies assessed the increase in the C_max_ and AUC and the decrease in the T_max_. In particular, a study reported that, after the intramuscular injection of CBD nanocrystals, the AUC and C_max_ of the CBD increased significantly compared with those obtained after the oral administration of both CBD nanocrystals and a CBD oily formulation [44].

Another pharmacokinetic study demonstrated that CBD in zein/whey protein nanoparticles given orally displayed 2- and 1.75-fold enhancements in the C_max_ and AUC, respectively, when compared with the free form of CBD [50]. In two further studies, the pharmacokinetics of SNEEDS were investigated and compared with oily formulations of CBD in rats to evidence the effects of the lipid components on the oral absorption. The studies evidenced that the effect of each type of lipid on the bioavailability of CBD is not straightforwardly anticipated. This unpredictable behaviour in vivo demonstrates the importance of investigating each vehicle preclinically following the in vitro development [52,53].

An interesting study evidenced that the AUC and C_max_ after the oral administration of CBD in an oily formulation were significantly reduced by 27- and 23-fold (*p* < 0.05 and *p* < 0.01, respectively), respectively, in bile-fistulated rats compared with the untreated rats. In contrast, all of the pharmacokinetic parameters after the oral administration of the CBD-loaded nanoemulsion were not significantly different between the untreated and bile-fistulated rats. Therefore, these results demonstrated that the conventional CBD oily formulation, but not CBD NE, requires bile-mediated micelle formation [54].

A study reported the bioavailability of liposomal formulations of CBD in an IRB-approved and monitored human crossover trial. The subjects received 10 mg oral CBD in either a naked or liposome formulation. The bioavailability of the CBD in the nanoformulation was ca. 17-fold greater than that of naked CBD after one hour of the oral administration (*p* < 0.05) [58].

The efficacy of the CBD-loaded nanoformulations was assessed in numerous studies. A study reported the in vitro antiproliferative properties of nanoparticles made of PLGA, and the detailed biological mechanism was described [49]. In addition, an in vivo veterinary trial in canine osteoarthritis assessed the efficacy of nanoliposomes loaded with CBD [46]. In a further study, nanostructured lipid particles were administered to mice orally and intranasally to cure neuropathic pain, and they were compared to a CBD oily formulation. Intranasally administered, the nanoparticles showed a better antinociception effect than the CBD oily formulation, both administered intranasally or orally. The nanoparticles also prolonged the period of therapeutic efficacy [57]. No side effects of the CBD loaded in the nanosystems were reported in the studies. Currently, there is a significant amount of safety evaluation data for CBD from preclinical and clinical studies, as reported by the database of the U.S. National Library of Medicine [61]. The World Health Organization’s report on CBD concluded that it has a good safety profile with limited side effects [62]. Three recent comprehensive reviews [63,64,65] primarily focused on the adverse effects and toxicity of CBD. Overall, the extensive preclinical and clinical studies evidenced that the risks associated with CBD at therapeutic doses (10–20 mg/kg bw/day) are considered acceptable by the FDA Center for Drug Evaluation and Research, and only liver toxicity is a potentially serious adverse health outcome. The most common side effects of Epidiolex are diarrhoea, headache, decreased appetite, and somnolence. In addition, CBD seems to enhance the rates of pneumonia compared with placebos, and high doses of CBD (≥20 mg/kg) have been associated with abnormal liver function tests, and particularly in patients taking the antiepileptic drug valproate concomitantly with Epidiolex. Overall, the incidence of side effects is low and, in comparison with other drugs, CBD has a better side effect profile.

**Figure 4:**
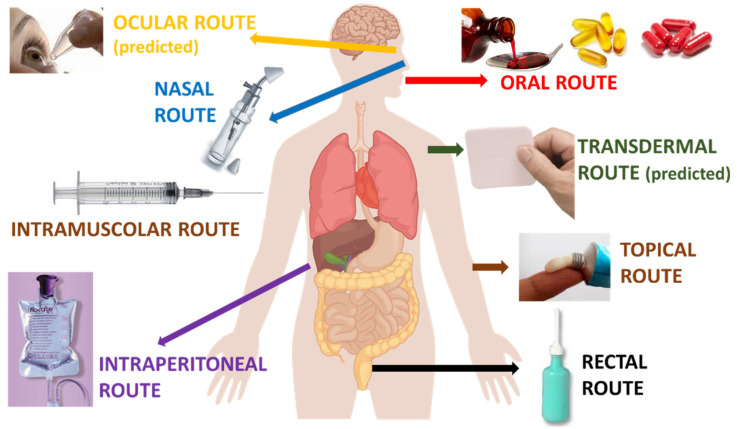
Tested or predicted routes of administration of the developed CBD-loaded nanocarriers.

Finally, the nanocarriers were successfully tested for their performance after the oral, parenteral, intranasal, topical, and colon-rectal administration routes. In addition, some of the nanoformulations were not directly tested but were developed for the transdermal and transcorneal routes of administration (see Figure 4).

## 6. Current Trends in Nanomedicines

Generally, a drug candidate’s efficacy, patient acceptability, and commercial feasibility largely depend on its delivery mode. Since the marketing of Doxil^®^ in 1995, nanomedicines have represented a superior therapeutic strategy compared with traditional formulations. To date, more than 100 nanomedicines are present on the market, and 563 are in the clinical process (phases I or II) or at other stages (663 in total). Most of these nanomedicines (53%) are anticancer drugs, followed by anti-infective drugs (14%), while the rest are drugs for the treatment of nervous system syndromes, blood, endocrine and metabolic illnesses, immunological disorders, inflammation, cardiovascular, ocular and skin diseases, and vaccines [66].

It is clear that nanocarriers dramatically modify the pharmacokinetics of a drug, with a consequent dramatic alteration in the therapeutic efficacy, and possibly also the safety profile. Therefore, these nanomedicines demand stringent evaluation. According to the international standard-setting body, the minimum set of measurements required by nanocarrier-based formulations includes the size, zeta potential, and solubility, which are considered significant predictors of their toxicity [67].

### 6.1. Marketing Authorisation of Nanomedicines

Nanomedicines are generally approved according to the conventional framework: the Food and Drug Administration (FDA), European Medicines Agency (EMA), and other agencies evaluate new medicines based on nanoparticles using a case-by-case approach under the traditional benefit/risk-analysis framework. In 2011, the European Commission defined the term “nanomaterial” as “a natural, incidental, or manufactured material containing particles, in an unbound state or as an aggregate or as an agglomerate and where, for 50% or more of the particles in the number size distribution, one or more external dimensions is in size range 1–100 nm” [68].

In 2006, the FDA’s Nanotechnology Task Force was established to issue the use of nanoscale materials to protect and promote public health [69]. “Guidance for Industry involving the Application of Nanotechnology, Liposome Drug Products: Chemistry, Manufacturing, and Controls; Human Pharmacokinetics and Bioavailability; and Labeling Documentation; Safety of Nanomaterials in Cosmetic Products; Use of Nanomaterials in Food for Animals; Drug Products, Including Biological Products, that Contain Nanomaterials” was published by the FDA [70].

The document reports on the potential risk factors for products containing nanomaterials, and it includes the quality attributes, structural and physicochemical characterisations, dissolution/in vitro drug release methods, safety, and stability. In addition, the absorption, distribution, metabolism, and excretion are considered. The risk considerations are related to the specific routes of administration and clinical development [70].

In the last two decades in Europe, only some “Recommendations” and “Reflection papers” have been developed concerning nanosized drug delivery systems [71]. To assist the pharmaceutical industry, the EMA published the first regulatory reflection paper on nanotechnology-based medicinal products for human use in 2006 [72].

In 2009, the EMA established the European Nanomedicines Expert Group, which is composed of high-profile academics and regulatory science specialists [73].

Up to now, the EMA has released four reflection papers related to block copolymer micelle products [74], and to the coating of nanomedicine products [75], as well as to intravenous liposomal products [76] and iron-based products [77], which were developed concerning innovator products.

Finally, in 2019, the EU signed mutual recognition agreements with third-country authorities (including the United States) concerning the conformity assessment of regulated products. Such agreements contain a sectoral annexe on the mutual recognition of good manufacturing practise inspections and the batch certification of human and veterinary medicines [78].

As for any medicinal product, the competent EU authorities are evaluating an application to place a nanomedicine product on the market, utilizing the established principles of benefit/risk analysis, rather than solely basing it on the technology per se [79].

### 6.2. Future Perspectives of Nanocarriers Loaded with CBD

The developed nanosystems to deliver CBD are very promising for a plethora of therapeutic opportunities, for their versatility in release and crossing physiological barriers, and for the different routes of administration.

According to the “Recommendations” and “Reflection papers” of the regulatory agencies, the simple architectures of nanocarriers (as conventional vesicles), and in particular, excipient-free nanomedicines (nanosuspensions), represent feasible directions to make the clinical translation easier. A significant challenge for developing nanodrugs is the characterisation of new nanomaterials concerning safety and toxicity.

Key points during the evaluation process by the regulatory agencies for the marketing authorisation are the route of administration, indication, function of the nanomaterial, structural complexity, and maturity of the technology (including the manufacturing processes, analytical techniques, and product design).

A rational evaluation of the studies reported in this review evidences how some of them are very preliminary [45,51,55,59,60]. Almost all the developed nanoparticles have simple architectures, are well known and safe nanocarriers, or are even simple nanosuspensions, and in addition, the conventional routes of administration are generally investigated. As a consequence, these studies are almost ready for forthcoming clinical translation [44,48,50,52,53,54,56,57,58].

In any case, the developed nanoformulations can provide unique solutions for the proper clinical practise of CBD, as proven by the numerous nanomedicines available on the market, and their in-depth development would be desirable.

## Figures and Tables

**Table 1:** Developed nanocarriers loaded with CBD and their compositions, CBD contents, CBD encapsulation efficiencies, and stability studies.

Type of Nanocarrier	Nanocarrier Composition	CBD Content	CBD Encapsulation Efficiency	Stability Studies	Reference
Nanosuspension	Tween 80	16.3% *w*/*w*	-	No data available	[44]
Nanosuspension	Lecithin HPMCAS Fe3O4	No data available	-	No data available	[45]
Nanomicelles	Pluronic F127	1% *w*/*w*	No data available	No data available	[46]
Nanomicelles	Chitosan PVA Poly(methyl methacrylate)	20% *w*/*w*	No data available	No data available	[47]
Nanoparticles	PLGA Chitosan	No data available	CBD–PLGA: 70.31 ± 0.69% CBD–CS–PLGA: 78.52 ± 0.82%	Physical and chemical stabilities at 4 °C after 5 weeks of storage (for both types of nanoparticles).	[48]
Nanoparticles	PLGA	1.5% *w*/*w*	95.23 ± 3.30%	Physical and chemical stabilities at 5 °C after three months of storage.	[49]
Nanoparticles	Zein Whey protein (WP)	Zein NP: 3.4% *w*/*w* Zein–WP NP: 0.6% *w*/*w*	Zein NP: 76% Zein–WP NP: 86%	Physical and chemical stabilities at 80 °C for 90 min, from UV irradiation, and at 4 °C after 3 weeks of storage.	[50]
Nanoemulsions	Poloxamer 188 Miglyol 812 (medium-chain triglycerides) Trimyristin Soybean oil Rapeseed oil	MCT-P188–120 nm: 73% *w*/*w* TM-P188–120 nm: around 70% *w*/*w* Soybean oil: 49% *w*/*w* Rapeseed oil: 45% *w*/*w*	Almost 100%	No data available	[51]
SNEEDS	Ethanol Soy phosphatidylcholine Sesame oil Tween 20 Span 80 Kolliphor RH40	3% *w*/*w*	Almost 100%	No data available	[52]
SNEEDS	Sesame oil Miglyol 812N Cocoa butter Tricaprin	Type I SNEDDS: 10% *w*/*w* Type II SNEDDS: 2% *w*/*w*	Almost 100%	No data available	[53]
Nanoemulsion	WaterVitamin E acetateEthanolTween 20	3% *w*/*w*	Almost 100%	No data available	[54]
Nanoemulsion	Water Soybean oil Quillaja saponin	1% *w*/*w*	Almost 100%	Physical and chemical stabilities at 4 °C after six weeks of storage. Stable at 80 °C for 1 min; at −20 °C for 1 h; after dilution of 1 mL to 355 mL of water or carbonated water. Tests with pH values less than 2, and salt concentrations greater than 100 mM, disrupted the nanoemulsion.	[55]
Microemulsion	Water Solutol HS 15 Transcutol P Isopropyl myristate	1% *w*/*w*	Almost 100%	Physical and chemical stabilities at 4 °C and 21 °C after three months of storage (both formulations).	[56]
Nanostructured lipid particles	Stearic acid Oleic acid Cetylpyridinium Chloride Span 20	18.75% *w*/*w*	99.99%	No data available	[57]
Nanovesicles	Sunflower lecithin	1 or 2% *w*/*w*	No data available	No data available	[58]
Nanovesicles	Soy lecithin Cholesterol Polysorbate 80	0.33% *w*/*w*	80.0 ± 0.077%	Physical and chemical stabilities at 25 °C after six months of storage.	[59]
Hybrid nanoparticles	Chitosan ZnO	No data available	No data available	No data available	[60]

**Table 2:** In vitro studies of the developed nanocarriers loaded with CBD.

Type of Nanocarrier	In Vitro Studies	Reference
Nanosuspension	Drug release	[44]
Nanosuspension	Drug release	[45]
Nanomicelles	Drug release MJ cells T-24 cells	[46]
Nanomicelles	hCEc cells	[47]
Nanoparticles	T-24 cells UM-UC-3 cells 5637 cells SVHUC-1 cells	[48]
Nanoparticles	Drug release SKOV-3	[49]
Nanoparticles	No data available	[50]
Nanoemulsions	No data available	[51]
SNEEDS	No data available	[52]
SNEEDS	No data available	[53]
Nanoemulsion	No data available	[54]
Nanoemulsion	No data available	[55]
Microemulsion	Drug release Ex vivo permeation (Franz cells) Skin-PAMPA	[56]
Nanostructured lipid particles	Drug release	[57]
Nanovesicles	RAW264.7 cells	[58]
Nanovesicles	Drug release Ex vivo permeation (Franz cells) Colorectal membrane integrity	[59]
Hybrid nanoparticles	Drug release L929 mouse fibroblast Water-vapor permeability	[60]

## Data Availability

Not applicable.
